# Cyclosporin A Promotes *in vivo* Myogenic Response in Collagen VI-Deficient Myopathic Mice

**DOI:** 10.3389/fnagi.2014.00244

**Published:** 2014-09-15

**Authors:** Francesca Gattazzo, Sibilla Molon, Valeria Morbidoni, Paola Braghetta, Bert Blaauw, Anna Urciuolo, Paolo Bonaldo

**Affiliations:** ^1^Department of Molecular Medicine, University of Padova, Padova, Italy; ^2^Interdepartmental Research Center E. Piaggio, University of Pisa, Pisa, Italy; ^3^Department of Biomedical Sciences, University of Padova, Padova, Italy

**Keywords:** collagen VI, skeletal muscle, congenital muscular dystrophy, animal model, cyclosporin A, muscle regeneration

## Abstract

Mutations of genes encoding for collagen VI cause various muscle diseases in humans, including Bethlem myopathy and Ullrich congenital muscular dystrophy. Collagen VI null (*Col6a1*^−/−^) mice are affected by a myopathic phenotype with mitochondrial dysfunction, spontaneous apoptosis of muscle fibers, and defective autophagy. Moreover, *Col6a1*^−/−^ mice display impaired muscle regeneration and defective self-renewal of satellite cells after injury. Treatment with cyclosporin A (CsA) is effective in normalizing the mitochondrial, apoptotic, and autophagic defects of myofibers in *Col6a1*^−/−^ mice. A pilot clinical trial with CsA in Ullrich patients suggested that CsA may increase the number of regenerating myofibers. Here, we report the effects of CsA administration at 5 mg/kg body weight every 12 h in *Col6a1*^−/−^ mice on muscle regeneration under physiological conditions and after cardiotoxin (CdTx)-induced muscle injury. Our findings indicate that CsA influences satellite cell activity and triggers the formation of regenerating fibers in *Col6a1*^−/−^ mice. Data obtained on injured muscles show that under appropriate administration, regimens CsA is able to stimulate myogenesis in *Col6a1*^−/−^ mice by significantly increasing the number of myogenin (MyoG)-positive cells and of regenerating myofibers at the early stages of muscle regeneration. CsA is also able to ameliorate muscle regeneration of *Col6a1*^−/−^ mice subjected to multiple CdTx injuries, with a concurrent maintenance of the satellite cell pool. Our data show that CsA is beneficial for muscle regeneration in *Col6a1*^−/−^ mice.

## Introduction

Collagen VI is an extracellular matrix protein that forms a microfibrillar network in the endomysium of skeletal muscle. The critical role played by this protein in muscle is clearly shown by collagen VI null (*Col6a1*^−/−^) mice, which display an early onset myopathic phenotype characterized by mitochondrial dysfunction, defective autophagy, and spontaneous apoptosis of muscle fibers (Bonaldo et al., 1998; Irwin et al., 2003; Grumati et al., 2010). Cyclosporin A (CsA) is a well-known immunosuppressant drug that was found to have multiple beneficial effects on the myopathic phenotype of *Col6a1*^-/-^ mice, including (i) decreased opening of the mitochondrial permeability transition pore; (ii) rescue of myofiber apoptosis; (iii) stimulation of autophagy in muscle fibers; and (iv) recovery of muscle strength (Irwin et al., 2003; Grumati et al., 2010). Mutations of *COL6* genes in humans cause several muscle disorders, including Bethlem myopathy and Ullrich congenital muscular dystrophy (Lampe and Bushby, 2005). A pilot clinical trial in Ullrich and Bethlem patients showed that CsA favorably affects mitochondrial function and dramatically decreases the incidence of apoptosis in muscle fibers. Notably, a significant increase in the number of regenerating myofibers was observed in younger patients undergoing CsA treatment, suggesting that CsA may also increase the overall efficiency of muscle regeneration in patients (Merlini et al., 2008, 2011).

Muscle regeneration relies on the presence of satellite cells, which are quiescent under physiological conditions but become activated upon damage, thus undergoing proliferation and terminal differentiation. At the same time, a subset of activated satellite cells returns to the quiescent state in their original niche under the basal lamina, through a self-renewal process (Tedesco et al., 2010). The differentiation of satellite cells is regulated by a number of transcription factors, where Pax7 is required for satellite cell specification and survival, whereas MyoD, myogenin (MyoG), and MRF4 are essential for satellite cell proliferation and differentiation (Buckingham and Rigby, 2014). Terminal differentiation coincides with the abundant synthesis of myosin heavy chain (MHC). The cardiotoxin (CdTx) injury model is widely used to investigate skeletal muscle regeneration (Chargé and Rudnicki, 2004; Shi and Garry, 2006). We recently demonstrated that collagen VI is a critical component of satellite cell *niche* and that ablation of collagen VI leads to impaired muscle regeneration and reduced satellite cell self-renewal after injury (Urciuolo et al., 2013). Studies performed in tibialis anterior (TA) muscle showed that *Col6a1*^−/−^ mice undergo a marked depletion of the satellite cell pool 7 days after CdTx injection, and this defect becomes much more dramatic after multiple rounds of CdTx injury (Urciuolo et al., 2013). Given the effects displayed by CsA in collagen VI-deficient mice and Ullrich/Bethlem patients (Irwin et al., 2003; Merlini et al., 2008), here we analyzed in detail the outcomes of CsA administration on muscle regeneration and satellite cells in *Col6a1*^−/−^ mice under physiological conditions and after CdTx-induced injury.

## Materials and Methods

### Mice

We performed experiments in wild-type mice of the inbred C57BL/6NCrl strain and in *Col6a1*^−/−^ mice that were backcrossed in the C57BL/6NCrl strain for eight generations (Irwin et al., 2003). All data were obtained from 6-month-old mice. Mice were housed in individual cages in an environmentally controlled room (23°C, 12 h light/12 h dark cycle) and provided food and water *ad libitum*. Mouse procedures were approved by the Ethics Committee of the University of Padova and authorized by the Italian Ministry of Health.

### *In vivo* treatments

Cyclosporin A (Sandimmun 50 mg/ml, Novartis) was dissolved in olive oil and a stock solution at a concentration of 10 mg/ml was prepared. For CsA administration under physiological conditions, mice were subjected to intraperitoneal (i.p.) injection of vehicle (olive oil) or CsA at 5 mg/kg body weight every 12 h for 10 days. In experiments with higher dosage CsA, mice were subjected to i.p. injection of vehicle or CsA at 25 mg/kg body weight every 24 h for 10 days. Animals were sacrificed 12 h after the last administration of CsA or vehicle. For single CdTx injury (Couteaux et al., 1988), mice were treated by i.p. injection with vehicle or CsA at 5 mg/kg body weight every 12 h for 10 days. At day 4 from the first administration of vehicle or CsA, mice were anesthetized with isoflurane (Merial) and TA muscles injected with 30 μl CdTx (Naja mossambica mossambica, 10 μM; Sigma). Analgesia (Rimadyl) was administered subcutaneously for 3 days and mice were sacrificed 7 days after muscle damage (i.e., 10 days after the first injection of vehicle or CsA). For multiple injury experiments, TA muscles were subjected to three distinct injections of CdTx, each one every 30 days. Four days before the third CdTx injection, mice were treated by i.p. injection with vehicle or CsA at 5 mg/kg body weight every 12 h for 10 days. Mice were sacrificed 30 days after the third CdTx injury (i.e., 24 days after the last injection of vehicle or CsA).

### Histological analysis

Tibialis anterior muscles were isolated from mice, frozen in liquid nitrogen, weighted on a precision balance, and kept at −80°C until use. Cross-sections (10 μm thick) were used and processed for hematoxylin–eosin or Azan-Mallory staining following standard protocols. Samples were analyzed with a Zeiss Axioplan light microscope equipped with Leica DC500 digital camera. Myofiber cross-sectional area and the area of fibrosis were evaluated with the IM1000 software (Leica).

### Isolation of extensor digitorum longus single myofibers

We carefully dissected extensor digitorum longus (EDL) muscles from 6-month-old mice and subjected them to enzymatic digestion with collagenase I (2 mg/ml, Gibco) for 80 min at 37°C. We blocked the digestion with Dulbecco’s Modified Eagle Medium (DMEM, Sigma), supplemented with 0.2 M l-glutamine (Invitrogen), 1:100 penicillin-streptomycin (Invitrogen), 1:100 fungizone (Invitrogen), and 10% horse serum (Gibco), and gently released single myofibers from muscles. Every 15–25 min, undamaged and non-contracted fibers were transferred in a new dish containing fresh medium, and this procedure was repeated five times in order to remove debris and interstitial cells. Freshly isolated fibers were finally fixed in 4% paraformaldehyde in PBS for 15 min and maintained at 4°C in PBS until use.

### Immunofluorescence

For immunofluorescence on muscle sections, frozen TA sections (7 μm) were fixed for 20 min with 4% paraformaldehyde in PBS and permeabilized for 6 min with cold methanol. For the unmasking of Pax7 and MyoG, slides were treated twice with 0.01 M citric acid (pH 6) at 90°C for 5 min. For mouse antibodies staining, samples were first incubated for 2.5 h with 4% bovine serum albumin (BSA IgG-Free, Jackson Immunoresearch) in PBS and then treated for 30 min with a blocking solution containing 0.05 mg/ml Fab fragment anti-mouse IgG (Jackson Immunoresearch). When mouse antibodies were not used, samples were only incubated for 1 h at room temperature with 4% bovine serum albumin in PBS. After the blocking step, samples were incubated with primary antibodies at 4°C overnight. The following primary antibodies were used: mouse anti-Pax7 (1:20; Developmental Studies Hybridoma Bank); mouse anti-MyoG (F5D, 1:15; Developmental Studies Hybridoma Bank); mouse anti-embryonic MHC (eMHC) (F1.652, 1:20; Developmental Studies Hybridoma Bank); rabbit anti-laminin (L9393, 1:800; Sigma). After washing, samples were incubated with the appropriate secondary antibody for 1 h at room temperature. Secondary antibodies used were biotinylated anti-mouse (115-007-003, 1:1000), Cy2 or Cy3 anti-mouse (115-226-062, 1:500, or 115-165-006, 1:1000), Cy2 or Cy3 anti-rabbit (111-225-144, 1:500, or 115-165-006, 1:1000) (all Jackson Immunoresearch). To reveal the biotinylated antibody, Cy2 or Cy3 streptavidin (016-220-084, 1:1500, or 016-160-084, 1:2500; Jackson Immunoresearch) was used. For immunofluorescence of EDL single myofibers, cells were permeabilized with 0.5% Triton X-100 in PBS, treated with 20% goat serum (Invitrogen) in PBS for 1 h, and incubated at 37°C for 1 h or at 4°C overnight with mouse anti-Pax7 antibody (1:20; Developmental Studies Hybridoma Bank). After washing, samples were incubated with the appropriate secondary antibody as described above. Nuclei were stained with Hoechst 33258 (Sigma). Samples were analyzed with a Zeiss Axioplan Leica DC500 epifluorescence microscope or with a Leica SP5 confocal microscope.

### TUNEL

For apoptosis analysis on TA cryosections, the DeadEnd™ Fluorometric TUNEL assay (Promega) was used. Samples were fixed for 15 min with 4% paraformaldehyde, permeabilized for 5 min with 0.5% Triton X-100, and processed following manufacturer instructions.

### Statistical analyses

Data are expressed as means ± SEM. We determined statistical significance by unequal variance Student’s *t*-test, and a *P* value of <0.05 was considered statistically significant.

## Results

### CsA induces muscle regeneration in *Col6a1*^−/−^ mice under physiological conditions

**Figure 1:**
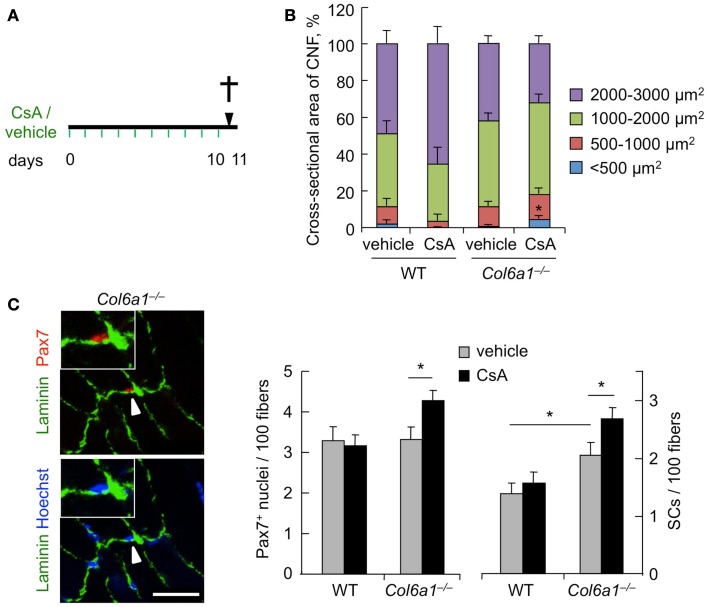
**Cyclosporin A induces muscle regeneration and increases satellite cell number in *Col6a1*^−/−^ mice**. **(A)** Schematic diagram of CsA treatment. Wild-type and *Col6a1*^−/−^ mice were treated with vehicle or with CsA (5 mg/kg body weight) every 12 h for 10 days. Animals were sacrificed 12 h after the last administration of CsA or vehicle. **(B)** Mean cross-sectional area of regenerating centrally nucleated myofibers in TA muscles derived from wild-type and *Col6a1*^−/−^ mice treated with vehicle or CsA. Fibers were divided into four size ranges, and at least 150 centrally nucleated myofibers were analyzed for each group. Error bars indicate SEM (*P < 0.05 for *Col6a1*^−/−^ CsA vs. *Col6a1*^−/−^ vehicle; *n* = 3–4, each group). **(C)** Left panel, representative images of immunofluorescence labeling for laminin (green) and Pax7 (red) in TA cross-sections of CsA-treated *Col6a1*^−/−^ mice. The arrowhead points at one satellite cell, shown at higher magnification in the inset. Nuclei were stained with Hoechst (blue). Scale bar, 25 μm. Right panel, quantification of total Pax7-positive cells and of satellite cells, calculated as the number on 100 myofibers in TA muscles derived from wild-type and *Col6a1*^−/−^ mice treated with vehicle or CsA. Error bars indicate SEM (*P < 0.05; *n* = 5–7, each group). CNF, centrally nucleated fibers; CSA, cross-sectional area; SCs, satellite cells; WT, wild-type.

To investigate the effects of CsA administration in *Col6a1*^−/−^ mice under physiological conditions, we subjected animals to i.p. injection of vehicle or CsA at 5 mg/kg body weight every 12 h and analyzed muscles after 10 days of treatment (Figure 1A). This dosage of CsA was previously found to trigger a marked amelioration of the myopathic phenotype of *Col6a1*^−/−^ mice, with rescue from mitochondrial depolarization and apoptosis and reactivation of the autophagic flux in muscle fibers (Irwin et al., 2003; Grumati et al., 2010). To evaluate whether this CsA treatment triggered *de novo* formation of myofibers in *Col6a1*^−/−^ mice, we first analyzed the cross-sectional area of regenerating, centrally nucleated fibers in TA muscle, by dividing regenerating myofibers into four different size ranges. Unlike wild-type animals, CsA treatment led to a significant increase of the percentage of regenerating myofibers with small area (<500 μm^2^) in *Col6a1*^−/−^mice when compared to vehicle-treated *Col6a1*^−/−^ animals (Figure 1B). These data were also confirmed by immunofluorescence analysis for eMHC, an established marker of newly forming fibers (Ciciliot and Schiaffino, 2010). Immature myofibers expressing eMHC were present in TA muscles of *Col6a1*^−/−^ mice treated with CsA, but not in those treated with vehicle (Figure S1A in Supplementary Material). Based on these results, we evaluated the number of myogenic cells by performing immunostaining for Pax7. CsA administration increased the total number of Pax7-positive cells and also the number of satellite cells (i.e., Pax7-positive cells located underneath the basal lamina) in *Col6a1*^−/−^ TA but not in wild-type TA (Figure 1C). These data were confirmed by analyzing freshly isolated EDL myofibers, which showed a significant increase in the number of Pax7-positive cells on myofibers derived from CsA-treated *Col6a1*^−/−^ mice when compared to vehicle-treated animals (Figure S1B in Supplementary Material). To assess whether the increased number of Pax7-positive cells in *Col6a1*^−/−^ animals was only provided by this drug dosage or could be also elicited by higher CsA concentrations known to cause strong immunosuppressive effects (Homan et al., 1980), we subjected mice to i.p. injection of CsA at 25 mg/kg body weight every 24 h for 10 days. Interestingly, at this higher dosage, CsA led to a dramatic decrease in the number of total Pax7-positive cells and of satellite cells both in wild-type and in *Col6a1*^−/−^ mice when compared to vehicle-treated animals (Figure S1C in Supplementary Material). These results highlight the relevance of CsA dosage in inducing beneficial effects in *Col6a1*^−/−^ muscles and accordingly with previous studies carried out with the immunosuppressive drug FK506 (Irwin et al., 2003), they suggest that immunosuppression exacerbates the phenotype of *Col6a1*^−/−^ mice.

### Continuous administration of CsA during CdTx injury stimulates the early phases of muscle differentiation in *Col6a1*^−/−^ mice

**Figure 2:**
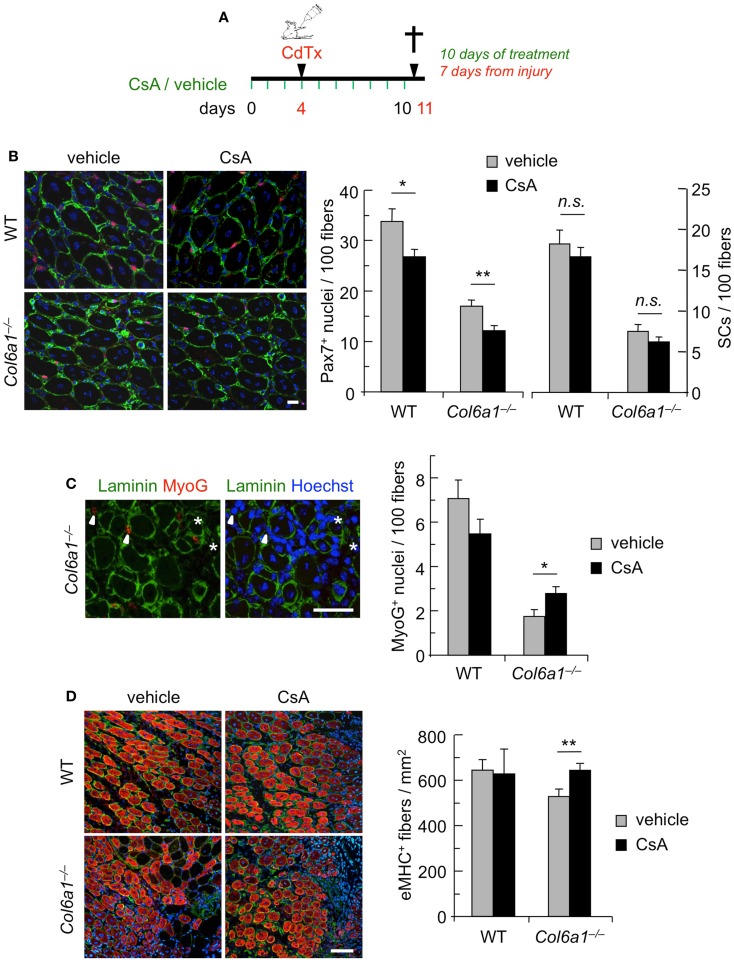
**Continuous administration of CsA during a single CdTx injury stimulates muscle differentiation in *Col6a1*^−/−^ mice**. **(A)** Schematic diagram of CsA treatment and CdTx injury. Wild-type and *Col6a1*^−/−^ mice were treated with vehicle or CsA (5 mg/kg body weight) every 12 h for 10 days. Four days after the first injection of vehicle or CsA, TA muscles were injured with CdTx and drug treatment continued for other 7 days. Animals were sacrificed 12 h from the last administration of CsA or vehicle. **(B)** Left panels, representative images of immunofluorescence labeling for laminin (green) and Pax7 (red) in 7-day-post-injury TA cross-sections of wild-type and *Col6a1*^−/−^ mice treated with vehicle or CsA. Scale bar, 25 μm. Right panel, quantification of total Pax7-positive cells and of satellite cells, calculated as the number on 100 myofibers in 7-day-post-injury TA muscles of wild-type and *Col6a1*^−/−^ mice treated with vehicle or CsA. Error bars indicate SEM (**P < 0.01; *P < 0.05; n.s. not significant; *n* = 3–5, each group). **(C)** Left panels, representative images of immunofluorescence labeling for laminin (green) and MyoG (red) in 7-day-post-injury TA cross-sections of *Col6a1*^−/−^ mice treated with vehicle or CsA. Nuclei were stained with Hoechst (blue). The arrowheads point at MyoG-positive cells outside the basal lamina, and the asterisks mark MyoG-positive cells located underneath the basal lamina (corresponding to myogenic cells that are undergoing fusion). Scale bar, 50 μm. Right panel, quantification of total MyoG-positive cells, calculated as the number on 100 myofibers in 7-day-post-injury TA muscles of wild-type and *Col6a1*^−/−^ mice treated with vehicle or CsA. Error bars indicate SEM (*P < 0.05; *n* = 3–5, each group). **(D)** Left panels, representative images of immunofluorescence for laminin (green) and eMHC (red) in 7-day-post-injury TA cross-sections of wild-type and *Col6a1*^−/−^ mice treated with vehicle or CsA. Nuclei were stained with Hoechst (blue). Scale bar, 50 μm. Right panel, quantification of the number of eMHC-positive myofibers per regenerating area in 7-day-post-injury TA cross-sections of wild-type and *Col6a1*^−/−^ mice treated with vehicle or CsA. Error bars indicate SEM (**P < 0.01; *n* = 3–5, each group). SCs, satellite cells; WT, wild-type.

To further assess the capability of CsA to ameliorate the muscle regenerative defects of *Col6a1*^−/−^ mice, we carried out CsA treatment under experimentally induced muscle injury. Toward this aim, wild-type and *Col6a1*^−/−^ mice were treated for 10 days with vehicle or CsA at 5 mg/kg body weight every 12 h; 4 days after the start of treatment, TA muscles were subjected to CdTx damage and mice were sacrificed 7 days after injury (Figure 2A). TUNEL assay showed that the incidence of apoptotic nuclei 7 days after CdTx injection was very low in wild-type TA, in agreement with the concept that myofiber demise is almost completed at this stage from injury (Hawke et al., 2003). Conversely, *Col6a1*^−/−^ TA showed a higher number of TUNEL-positive myonuclei 7 days after injury, and CsA administration was able to significantly decrease the incidence of apoptotic myofibers triggered by CdTx injury in *Col6a1*^−/−^ TA muscles (Figure S2 in Supplementary Material). We next evaluated the number of myogenic cells by immunofluorescence for the Pax7 and MyoG markers. CsA administration led to a significant decrease in the number of total Pax7-positive cells in both wild-type and *Col6a1*^−/−^ injured TA, without any significant change in the number of satellite cells (i.e., Pax7-positive cells located underneath basal lamina) (Figure 2B). This response was paralleled by a significant increase of the total number of MyoG-positive cells in injured TA muscles of CsA-treated *Col6a1*^−/−^ mice when compared to vehicle-treated *Col6a1*^−/−^ mice, whereas wild-type injured TA muscles did not show any significant difference in MyoG positivity between vehicle and CsA-treated animals (Figure 2C). Additionally, *Col6a1*^−/−^ mice treated with CsA showed an increased number of regenerating myofibers expressing eMHC, whereas no differences in eMHC-positive regenerating myofibers were found in wild-type animals (Figure 2D). As the defective satellite self-renewal of *Col6a1*^−/−^ mice is strictly dependent on the lack of extracellular collagen VI and on the lower muscle stiffness (Urciuolo et al., 2013), it was not surprising to observe that in this experimental condition CsA does not display any overt effect on satellite cell maintenance. On the other hand, the remarkable increase in the number of differentiated (i.e., MyoG-positive) myogenic cells, together with the higher number of newly forming (i.e., eMHC-positive) myofibers, indicates that CsA administration is able to improve muscle differentiation upon damage in the *Col6a1*^−/−^ myopathic mouse model.

### Administration of CsA during repeated muscle injury counteracts muscle loss and fibrosis and preserves the satellite cell pool in *Col6a1*^−/−^ mice

**Figure 3:**
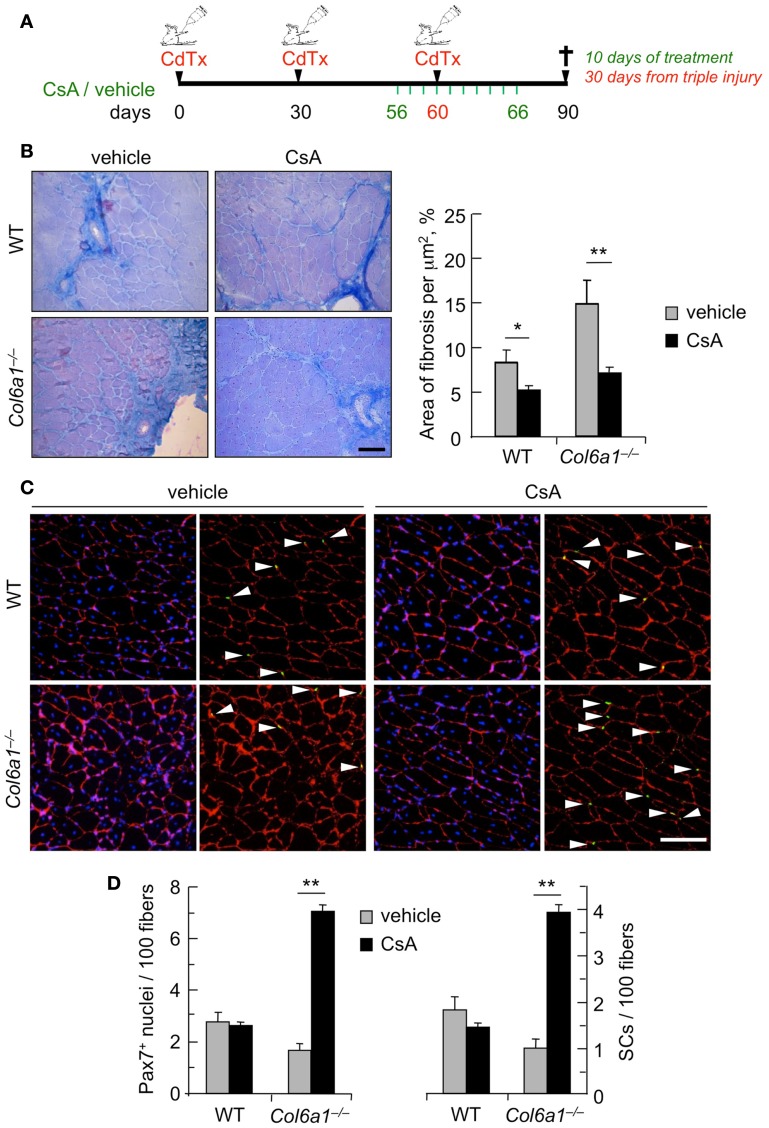
**Administration of CsA during a triple muscle injury counteracts muscle loss and fibrosis and preserves the satellite cell pool in *Col6a1*^−/−^ mice**. **(A)** Schematic diagram of CsA treatment and CdTx injuries. TA muscles of wild-type and *Col6a1*^−/−^ mice were given three repeated injections of CdTx every 30 days. Four days before the last injury, *Col6a1*^−/−^ mice were treated with vehicle or CsA (5 mg/kg body weight) every 12 h for 10 days. Mice were sacrificed 30 days after the third injury (i.e., 24 days from the last administration of vehicle or CsA). **(B)** Left panels, Azan-Mallory staining of triple injured TA cross-sections from wild-type and *Col6a1*^−/−^ mice treated with vehicle or CsA. Scale bar, 50 μm. Right panel, quantification of the fibrotic area in triple-injured TA cross-sections from wild-type and *Col6a1*^−/−^ mice treated with vehicle or CsA. Error bars indicate SEM (**P < 0.01; *P < 0.05; *n* = 4–8, each group). **(C)** Representative images of immunofluorescence labeling for laminin (red) and Pax7 (green) in triple-injured TA cross-sections from wild-type and *Col6a1*^−/−^ treated with vehicle or CsA. Arrows point at Pax7-positive nuclei. Nuclei were stained with Hoechst (blue). Scale bar, 100 μm. **(D)** Quantification of total Pax7-positive cells and of satellite cells, calculated as the number on 100 myofibers in triple-injured TA cross-sections from wild-type and *Col6a1*^−/−^ mice treated with vehicle or CsA. Error bars indicate SEM (**P < 0.01; *n* = 4–8, each group). SC, satellite cells; WT, wild-type.

Although muscles of *Col6a1*^−/−^ animals display a delayed regeneration after injury, we have previously shown that at 30 days after CdTx injury they are still able to complete the regeneration process (Urciuolo et al., 2013). However, and at difference from wild-type mice, the capability of *Col6a1*^−/−^ animals to undergo muscle regeneration and preserve the satellite cell pool is lost after multiple muscle injuries, leading to a severe loss of muscle mass (Urciuolo et al., 2013). Therefore, we investigated whether CsA is able to counteract the defective muscle regeneration and the depletion of the satellite cell pool triggered by multiple injuries in *Col6a1*^−/−^ mice. Toward this aim, we subjected TA muscles of wild-type and *Col6a1*^−/−^ mice to three rounds of CdTx injury. Animals were treated for 10 days with vehicle or CsA at 5 mg/kg body weight every 12 h during the third injury and sacrificed 30 days after the last injury (Figure 3A). Interestingly, CsA administration was highly effective in reducing the extensive muscle fibrosis triggered by triple injury in *Col6a1*^−/−^ mice (Figure 3B). The beneficial effects of CsA in *Col6a1*^−/−^ muscles undergoing multiple injuries were also confirmed by the increased myofiber cross-sectional area and by the improvement of the muscle mass in CsA-treated *Col6a1*^−/−^ mice when compared to vehicle-treated *Col6a1*^−/−^ mice (Figure S3 in Supplementary Material). Notably, CsA administration led to marked increase of both total Pax7-positive cell number and satellite cell number in *Col6a1*^−/−^ TA muscles subjected to multiple injuries (Figures 3C,D). Altogether, these results show that at this regimen CsA is capable to preserve not only muscle fibers but also the satellite cell pool of collagen VI-deficient mice.

## Discussion

In the present study, we evaluated the potential beneficial effects exerted by CsA on skeletal muscle regeneration in *Col6a1*^−/−^ mice, both under physiological condition and after muscle damage. The rationale for this study was based on previous findings in patients affected by collagen VI myopathies, suggesting that besides counteracting myofiber apoptosis and mitochondrial dysfunction, CsA treatment may also increase muscle regeneration (Merlini et al., 2008, 2011). In addition, our recent findings showed impaired regeneration and defective satellite cell self-renewal in collagen VI-deficient muscles (Urciuolo et al., 2013). Therefore, we investigated how CsA treatment impacts on the regeneration of collagen VI-deficient mice. Under physiological conditions, CsA was capable to amplify the pool of total Pax7-positive cells and of satellite cells and increased the amount of newly formed centrally nucleated myofibers, thus suggesting a new role for CsA in stimulating myogenesis in *Col6a1*^−/−^ muscles. Notably, these beneficial effects were dose-dependent, as they were observed at 10 mg/kg/day but not at a higher immunosuppressant dose (25 mg/kg/day), which conversely had a negative impact on the satellite cell pool. These data are in agreement with previous results in which the same protocol of CsA administration was found to desensitize the mitochondrial permeability transition pore and reduce myofiber apoptosis in *Col6a1*^−/−^ mice (Irwin et al., 2003). Although we previously demonstrated that CsA stimulates autophagy in skeletal myofibers (Grumati et al., 2010), the increased number of satellite cells in muscles of CsA-treated *Col6a1*^−/−^ animals is not a direct consequence of a stimulatory effect on autophagy. Indeed, reactivation of the autophagic flux in *Col6a1*^−/−^ mice by different pharmacological or dietary treatments does not exert any significant effect on satellite cells (Urciuolo et al., 2013). Our present findings are consistent with the strong amelioration of the myophatic phenotype in *Col6a1*^−/−^ mice following CsA administration and indicate that besides decreasing mitochondrial dysfunction and apoptosis and reactivating autophagy in muscle fibers (Irwin et al., 2003; Grumati et al., 2010), the drug is also able to increase the pool of functional Pax7-positive cells and stimulate the formation of newly formed fibers.

The beneficial effects exerted by CsA on the regeneration capabilities of *Col6a1*^−/−^ mice become very evident under experimentally induced single and multiple muscle injuries. Our data indicate that CsA is capable to induce myogenesis in *Col6a1*^−/−^ mice after muscle damage. In fact, when TA muscles were damaged during a continuous CsA administration, analysis at 7 days post-injury showed that CsA elicits a significant increase in the number of MyoG-positive cells and of regenerating myofibers in *Col6a1*^−/−^ muscles. Interestingly, this response is not associated with an improvement of the number of satellite cells, suggesting that under these conditions CsA is unable to ameliorate satellite cell self-renewal. The effect of CsA on muscle regeneration was even more remarkable when we exacerbated the muscle phenotype of *Col6a1*^−/−^ mice through triple CdTx damage. Our findings indicate that CsA is protective against fibrotic tissue formation, maybe exerting this effect through an indirect regulation of the inflammatory state that occurs during muscle regeneration (Serrano et al., 2011). A similar beneficial effect of CsA in reducing muscle fibrosis was reported for *mdx* mice undergoing exercise (De Luca et al., 2005). Furthermore, CsA administration was able to counteract the loss of satellite cells elicited by repeated muscle injuries in *Col6a1*^−/−^ animals, concurrently guaranteeing myogenic differentiation, as confirmed by the increase of myofiber cross-sectional area and muscle mass. Although it was beyond the scope of this study to dissect the mechanism(s) through which CsA leads to increased satellite cell number in *Col6a1*^−/−^ mice after repeated injuries, it can be hypothesized that CsA administration may not directly influence the self-renewal capability of satellite cells and that the preservation of satellite cell pool may be mediated by an increase of their survival. This assumption is supported by the fact that the defective satellite cell self-renewal of *Col6a1*^−/−^ mice is strictly dependent on the lack of collagen VI itself and its consequences on muscle stiffness (Urciuolo et al., 2013) and that CsA treatment is able to reduce apoptosis in *Col6a1*^−/−^ muscles (Irwin et al., 2003). To our knowledge, no literature work has investigated in detail the effects of *in vivo* CsA administration on stem cell homeostasis in skeletal muscles. A recent study reported some beneficial effects of CsA on neuronal stem cells, showing that *in vivo* CsA administration increases the number of neurospheres due to enhanced neuronal stem cell survival, rather than increased proliferation (Hunt et al., 2010).

The pharmacology of CsA is complex, and the drug binds a family of cellular peptidyl-prolyl *cis–trans* isomerases known as cyclophilins. Binding of CsA with the abundant cyclophilin A leads to inhibition of calcineurin, a cytosolic phosphatase found in many cell types, thus preventing dephosphorylation of its substrates (Liu et al., 1991). A number of studies have shown that calcineurin signals are involved in the control of myofiber size, myofiber type, and skeletal muscle regeneration (Schiaffino and Serrano, 2002; Sakuma and Yamaguchi, 2010; Hudson and Price, 2013). Although inhibition of calcineurin was shown to delay muscle regeneration (Sakuma et al., 2003, 2005), literature studies investigating the outcomes of calcineurin inhibition by genetic approaches or by CsA administration in animal models of muscle diseases have produced contrasting results (Stupka et al., 2004; De Luca et al., 2005; Parsons et al., 2007). The reasons for these discrepancies rely upon multiple factors, including the genetic model studied, the dose of the drug, the type of muscle, the duration of treatment, and the route of treatment. For instance, the efficacy of CsA in the *mdx* mice, an animal model of Duchenne muscular dystrophy, was reported to be dependent on the dosage and length of the treatment (Stupka et al., 2004; De Luca et al., 2005). Notably, the protective effects of CsA in *Col6a1*^−/−^ mice do not rely upon calcineurin inhibition, as the same beneficial effects are also displayed by non-immunosuppressive CsA analogs that do not bind calcineurin, such as Debio 025 and NIM811 (Angelin et al., 2007; Zulian et al., 2014), whereas they cannot be mimicked by the calcineurin inhibitor FK506 (Irwin et al., 2003). Although our interest was far from the study of calcineurin activity, in this work we used a definite CsA dosage (5 mg/kg every 12 h, i.e., the same dose shown to be effective in rescuing different aspects of the muscle pathology of *Col6a1*^−/−^ mice), and this dosage is known to only partially reduce the activity of calcineurin (Dunn et al., 2002; Michel et al., 2004).

In conclusion, our results indicate that besides the already known beneficial effects of CsA administration in ameliorating the myophatic phenotype of *Col6a1*^−/−^ mice through the rescue from mitochondrial and autophagic dysfunction of muscle fibers, CsA is also capable to stimulate muscle regeneration and preserve the satellite cell pool in this disease model. These findings support and strengthen the increased muscle regeneration observed in Ullrich patients undergoing clinical trial with CsA, pointing at CsA and its non-immunosuppressive derivatives as a promising therapeutic route for this group of inherited muscle diseases.

## Author Contributions

Francesca Gattazzo planned and performed *in vivo* and *ex vivo* experiments and wrote the paper. Sibilla Molon performed cardiotoxin damage, immunofluorescence, and histology. Valeria Morbidoni performed *in vivo* satellite cell quantification. Bert Blaauw carried out part of the *in vivo* studies. Paola Braghetta was involved in CsA administration. Anna Urciuolo oversaw the results and interpreted the data. Paolo Bonaldo oversaw the results and wrote the paper. All the authors discussed the results, revised the work, commented on the manuscript, and agreed on the final draft.

## Conflict of Interest Statement

The authors declare that the research was conducted in the absence of any commercial or financial relationships that could be construed as a potential conflict of interest.

## Supplementary Material

The Supplementary Material for this article can be found online at http://www.frontiersin.org/Journal/10.3389/fnagi.2014.00244/abstract
