# Supplementary material for: Cyclosporin A Promotes in vivo Myogenic Response in Collagen VI-Deficient Myopathic Mice
Source: Front Aging Neurosci. 2014 Sep 15;6:244. doi: 10.3389/fnagi.2014.00244 (PMC4163991; doi:10.3389/fnagi.2014.00244)
Supplement: Supplementary file 1 [file Data_Sheet_1.PDF]

SUPPLEMENTARY MATERIAL

Cyclosporin A promotes *in vivo* myogenic response in collagen VI deficient myopathic mice

Francesca Gattazzo[1,2], Sibilla Molon[1], Valeria Morbidoni[1], Paola Braghetta[1], Bert Blaauw[3], Anna Urciuolo[1,#,*], and Paolo Bonaldo[1*]

[1] Department of Molecular Medicine, University of Padova, 35131 Padova, Italy
[2] Interdepartmental Research Center "E. Piaggio", University of Pisa, 56126 Pisa, Italy
[3] Department of Biomedical Sciences, University of Padova, 35131 Padova, Italy

*** Correspondence:** Paolo Bonaldo, Department of Molecular Medicine, University of Padova, Via U. Bassi 58/B, I-35131 Padova, Italy. e-mail: bonaldo@bio.unipd.it; Anna Urciuolo, Department of Molecular Medicine, University of Padova, Via U. Bassi 58/B, I-35131 Padova, Italy. e-mail: a.urciuolo@ucl.ac.uk

[#] **Present address:** Anna Urciuolo, Stem Cells & Regenerative Medicine, University College London Institute of Child Health, London WC1N 1EH, United Kingdom.

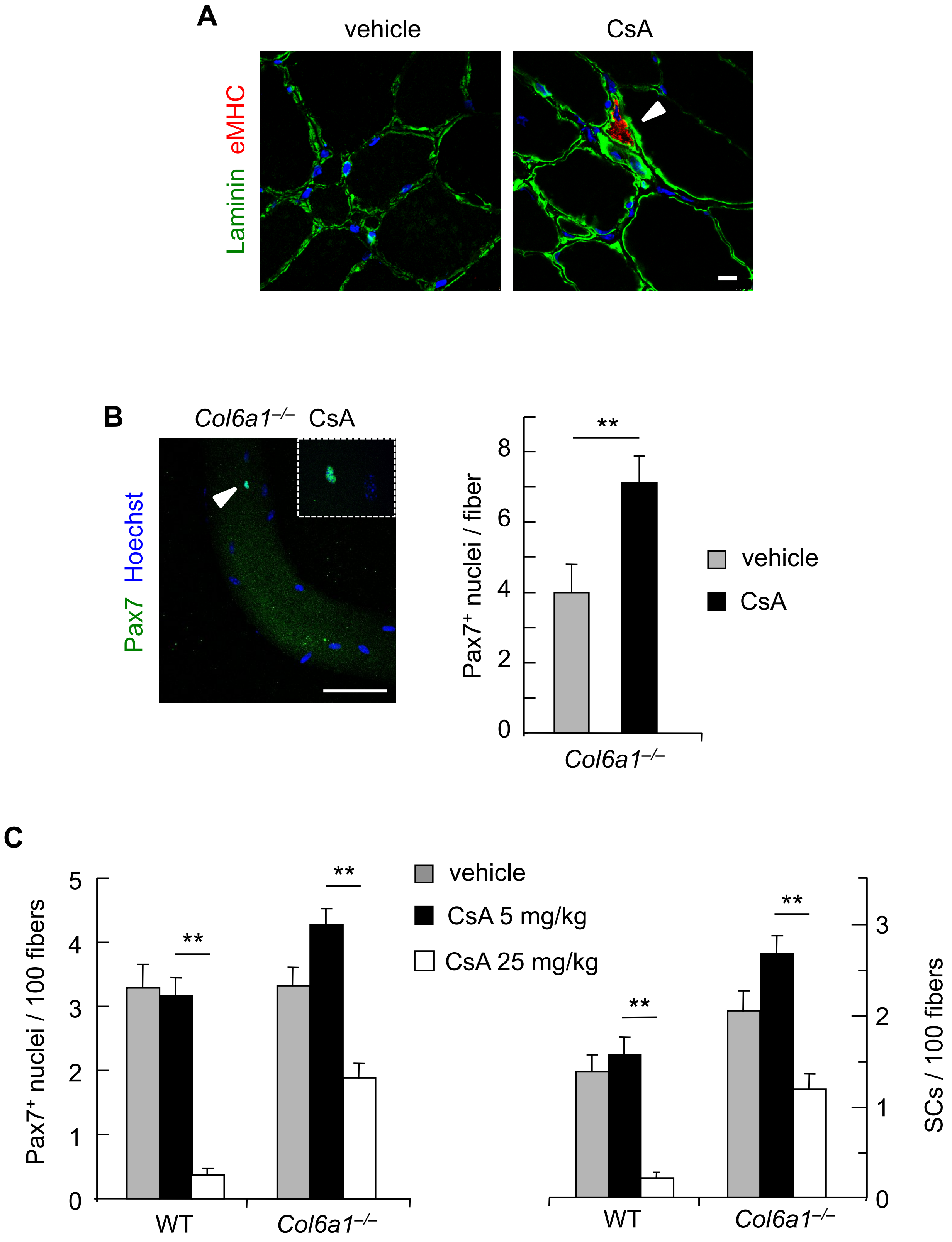

Supplementary Figure S1

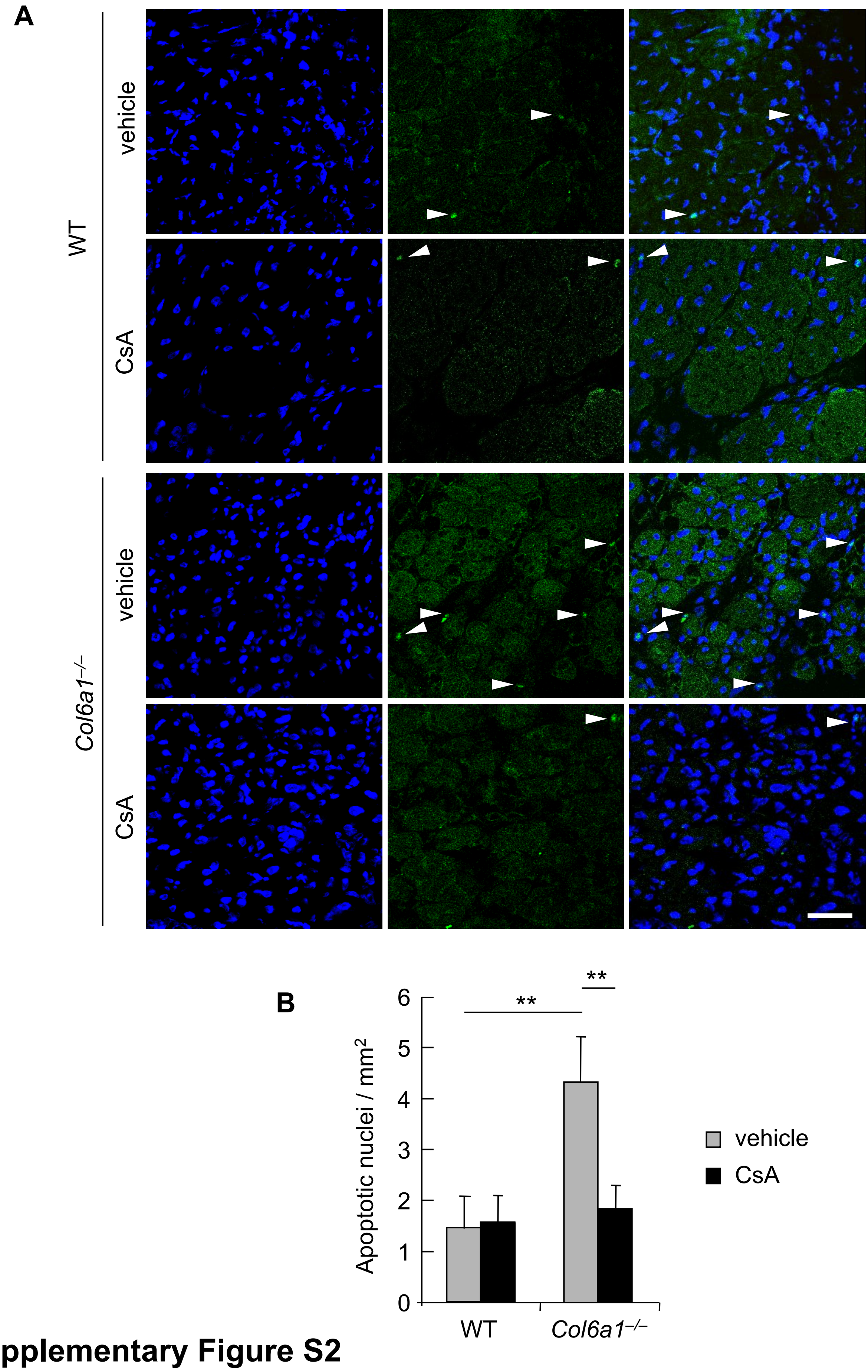

Supplementary Figure S2

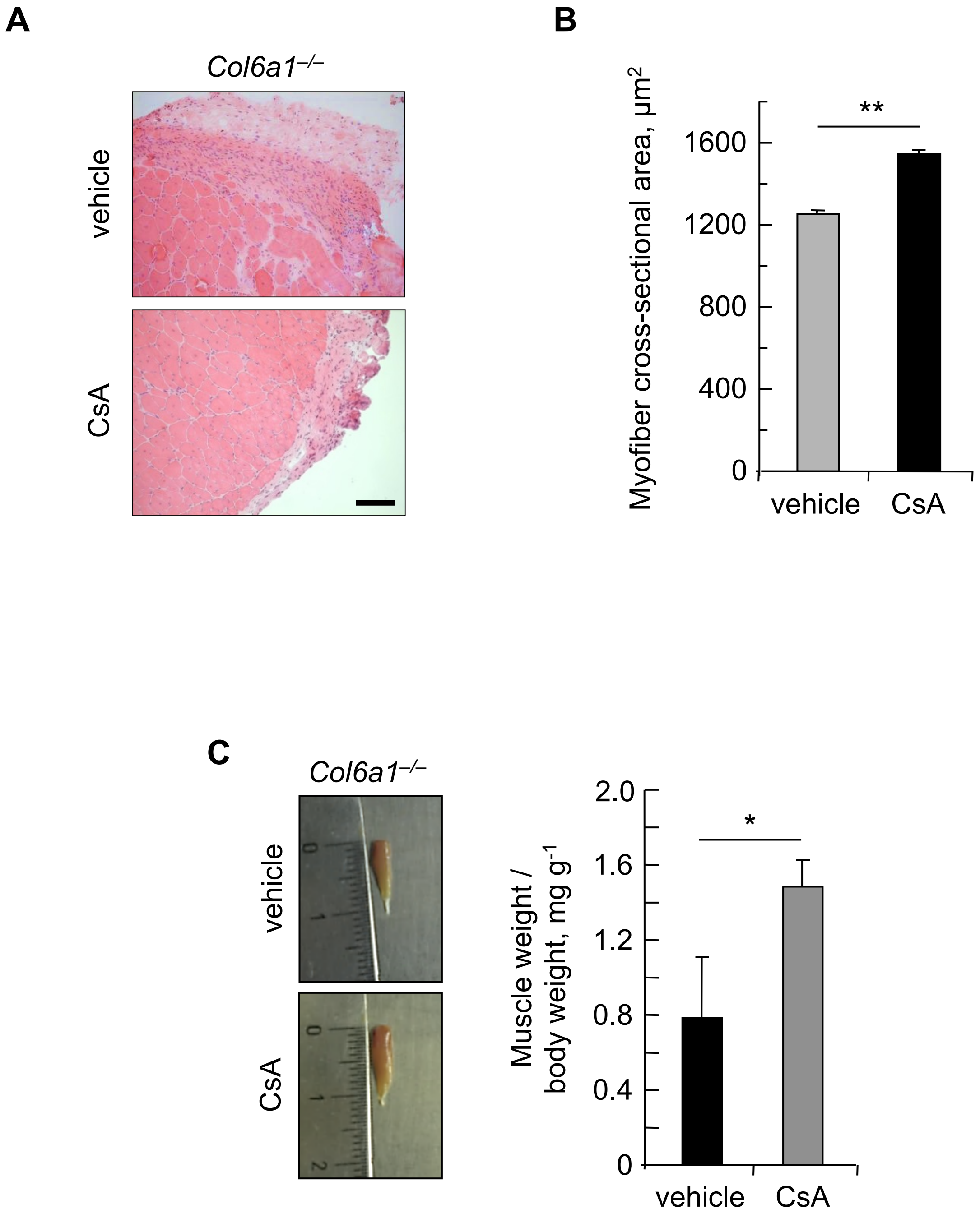

Supplementary Figure 3

Supplementary Figure Legends

Supplementary Figure S1. CsA treatment induces muscle regeneration and increases satellite cell number in *Col6a1*$^{-/-}$ muscles. (**A**) Representative images of immunofluorescence for eMHC (red) and laminin (green) in TA cross-sections of *Col6a1*$^{-/-}$ mice treated with vehicle or CsA. The arrowhead points at one eMHC-positive myofiber. Scale bar, 10 µm. (**B**) Left panel, representative image of immunofluorescence labeling for Pax7 (green) in freshly isolated single EDL muscle fibers derived from CsA-treated *Col6a1*$^{-/-}$ mice. A Pax7-positive cell (arrowhead) is shown at higher magnification in the inset. Nuclei were stained with Hoechst (blue). Scale bar, 75 µm. Right panel, quantification of Pax7-positive nuclei in freshly isolated single EDL fibers derived from *Col6a1*$^{-/-}$ mice treated with vehicle or CsA. Error bars indicate s.e.m. (*$P < 0.05$; n = 8-9, each group). **(C)** Quantification of total Pax7-positive cells and of satellite cells, calculated as the number on 100 myofibers, in wild-type and *Col6a1*$^{-/-}$ TA muscles derived from mice treated for 10 days with vehicle or with CsA at 5 mg/kg every 12 hr or at 25 mg/kg every 24 hr. Error bars indicate s.e.m. (** $P <$ 0.01; n = 5-7, each group). SCs, satellite cells; WT, wild-type.

Supplementary Figure S2. Analysis of apoptosis in 7-day-post-injury TA muscles from wild-type and *Col6a1*$^{-/-}$ mice. (**A**) Representative images of TUNEL analysis in TA cross-sections derived from wild-type and *Col6a1*$^{-/-}$ mice treated for 10 days with vehicle or CsA and sacrificed 7 days after CdTx injury. Arrowheads point at TUNEL-positive nuclei (green). Nuclei were stained with Hoechst. Scale bar, 75 µm. (**B**) Quantification of TUNEL-positive nuclei in 7-day-post-injury TA muscles of wild-type and *Col6a1*$^{-/-}$ mice treated with vehicle or CsA. Error bars indicate s.e.m. (** $P < 0.05$; n = 3-5, each group). WT, wild-type.

Supplementary Figure S3. CsA treatment ameliorates muscle regeneration in *Col6a1*$^{-/-}$ mice undergoing multiple CdTx injury. (**A**) Haematoxylin-eosin staining of triple injured TA cross-sections from *Col6a1*$^{-/-}$ mice treated with vehicle or CsA. Scale bar, 50 µm. (**B**) Mean myofiber cross-sectional area in triple injured TA cross-sections from *Col6a1*$^{-/-}$ mice treated with vehicle or CsA. Error bars indicate s.e.m. (** $P < 0.01$; n = 4-8, each group). (**C**) Left panels, representative images of triple injured whole TA muscle dissected from *Col6a1*$^{-/-}$ mice treated with vehicle or CsA. Right panel, quantification of TA weight per body weight 30 days post-triple injury in *Col6a1*$^{-/-}$ mice treated with vehicle or CsA. Error bars indicate s.e.m. (* $P < 0.05$; n = 4-8, each group).
